# Final results of a phase 1b study of isatuximab short-duration fixed-volume infusion combination therapy for relapsed/refractory multiple myeloma

**DOI:** 10.1038/s41375-021-01262-w

**Published:** 2021-05-28

**Authors:** Saad Z. Usmani, Chatchada Karanes, William I. Bensinger, Anita D’Souza, Noopur Raje, Sascha A. Tuchman, Douglas Sborov, Jacob P. Laubach, Giada Bianchi, Dheepak Kanagavel, Rao Saleem, Franck Dubin, Frank Campana, Paul G. Richardson

**Affiliations:** 1 Levine Cancer Institute/Atrium Health, Charlotte, NC USA; 2 City of Hope National Medical Center, Duarte, CA USA; 3 Myeloma & Transplant Program, Swedish Cancer Institute, Seattle, WA USA; 4 Medical College of Wisconsin, Milwaukee, WI USA; 5 Massachusetts General Hospital, Boston, MA USA; 6 Division of Hematology, University of North Carolina at Chapel Hill, Chapel Hill, NC USA; 7 Division of Hematology & Hematologic Malignancies, University of Utah, Salt Lake City, UT USA; 8 Department of Medical Oncology, Dana-Farber Cancer Institute, Harvard Medical School, Boston, MA USA; 9 Sanofi R&D, Vitry-Alfortville, France; 10 Sanofi, Cambridge, MA USA; 11 Present Address: Takeda Pharmaceuticals, Cambridge, MA USA

**Keywords:** Myeloma, Drug development

## Abstract

Part B of this phase 1b study (ClinicalTrials.gov number, NCT02283775) evaluated safety and efficacy of a fixed-volume infusion of isatuximab, an anti-CD38 monoclonal antibody, in combination with pomalidomide and dexamethasone (Pd) in relapsed/refractory multiple myeloma patients. Isatuximab (10 mg/kg weekly for 4 weeks, then every other week) was administered as a fixed-volume infusion of 250 mL (mL/h infusion rate) with standard doses of Pd on 28-day cycles. Patients (*N* = 47) had a median of three prior treatment lines (range, 1–8). Median duration of exposure was 36.9 weeks and median duration of first, second, and 3+ infusions were 3.7, 1.8, and 1.2 h, respectively. The most common non-hematologic treatment-emergent adverse events were fatigue (63.8%), infusion reactions (IRs), cough, and upper respiratory tract infection (40.4% each). IRs were all grade 2 and occurred only during the first infusion. The overall response rate was 53.2% in all patients (55.5% in response-evaluable population, 60.0% in daratumumab-naïve patients). Efficacy and safety findings were consistent with data from the isatuximab plus Pd infusion schedule in Part A of this study and also from the phase 3 ICARIA-MM study, and these new data confirm the safety, efficacy, and feasibility of fixed-volume infusion of isatuximab.

## Introduction

Multiple myeloma (MM) is a plasma cell disorder characterized by the excess production of a monoclonal immunoglobulin protein causing end-organ damage [1, 2]. The disease is considered incurable and the majority of patients will relapse during their lifetime, thus long-term disease control is the pragmatic goal for most MM patients [1, 3]. Proteasome inhibitors (PIs), immunomodulatory drugs (IMiDs), and autologous stem cell transplantation have extended overall survival [3–5], however, the majority of patients will develop disease that is refractory to these treatments and the prognosis of relapsed/refractory MM (RRMM) patients remains poor [6, 7].

Recently, monoclonal antibodies have dramatically improved the MM treatment landscape [2]. CD38 is a surface antigen that is abundantly expressed on plasma cells, making it an attractive MM therapeutic target [8–10]. Isatuximab, a monoclonal, anti-CD38 antibody that targets a unique CD38 epitope, has antitumor activity through multiple mechanisms of action, including antibody-dependent cellular-mediated cytotoxicity, complement-dependent cytotoxicity, antibody-dependent cellular phagocytosis, and direct induction of apoptosis [9, 11, 12]. Furthermore, isatuximab inhibits CD38 ADP ribosyl-cyclase enzymatic activity, altering the immunotolerant bone marrow milieu [13]. Finally, isatuximab also induces indirect antitumor activity through the elimination of CD38^+^ immunosuppressive Treg cells [9] and an “in vivo vaccination” effect against CD38 as well as other MM-associated antigens [14].

Isatuximab has shown promise in treatment of MM as monotherapy and in combination with other therapies [15–19]. To date, isatuximab (Sarclisa^®^) has been approved in the United States, Europe, Switzerland, Canada, Australia, Japan, and Russia for use in combination with pomalidomide and dexamethasone (Pd) to treat adults with RRMM who have received at least two prior therapies, including lenalidomide and a PI [20–23]. While the addition of pomalidomide to dexamethasone improves survival in patients with MM [24], long-term outcomes with this regimen remain poor for patients who are heavily pretreated or who have refractory disease [4]. Isatuximab enhances the anti-MM activity of standard therapies, including IMiDs like pomalidomide [25] and IMiDs plus anti-CD38 therapies have been shown to lengthen progression-free survival (PFS) [26, 27]. The pivotal phase 3 ICARIA-MM study (ClinicalTrials.gov number, NCT02990338) compared treatment of isatuximab plus Pd (Isa-Pd) with Pd alone [6, 28]. The addition of isatuximab to Pd resulted in a significant and clinically meaningful benefit in PFS in heavily treated patients with RRMM with a manageable safety profile.

The potential for infusion reactions (IRs) with isatuximab requires premedication and careful attention to infusion rates. This phase 1b, open-label, multicenter, non-comparative study (ClinicalTrials.gov number, NCT02283775) [29] evaluated Isa-Pd in patients with RRMM who had been treated with lenalidomide and a PI. Part A was a dose escalation to assess the safety and efficacy of Isa-Pd in 45 patients with a median of three prior lines of treatment including lenalidomide and a PI [29]. Patients received isatuximab at 5, 10, or 20 mg/kg in four weekly doses (cycle 1), and every other week after. Initial isatuximab infusion rate was 87.5 mg/h for 5 mg/kg cohort and 175 mg/h for the 10 and 20 mg/kg cohorts. Every 30 min, infusion rate increased by 50 mg/h (first infusion) or 100 mg/h (subsequent infusions) to a maximum of 400 mg/h, in the absence of IRs. In an expansion cohort, 22 patients were treated at the selected dose of 10 mg/kg. To allow for a reduction in the infusion time, the infusion rate (mg/hour) was increased following the first infusion. The median infusion time for the first infusion was 3.3 h and 2.9 h for subsequent infusions. Of the IRs reported (42% of patients), all were grade 1/2 in severity, except for one grade 3 IR. The majority of IRs occurred during the first infusion (42%), with 6.7% occurring with later infusions. The combination of isatuximab with Pd was well tolerated by heavily pretreated patients with RRMM. The overall response rate (ORR) was 64.5% for Isa-Pd and median PFS was 17.6 months (95% confidence interval [CI], 6.8–20.5).

The goal of Part B (reported here) is to evaluate the feasibility and safety of fixed-volume infusion of isatuximab 10 mg/kg plus Pd in patients with RRMM. We hypothesized that a one-step infusion process using a fixed-volume infusion (expressed in mL/h) could have a similarly favorable efficacy and safety profile as was demonstrated with the infusion volume based on patient weight used in Part A, while reducing the duration of infusions.

## Methods

This was a multicenter, open-label, non-comparative, phase 1b study comprised of two parts, of which Part A has been previously described [29]. Eligible patients were at least 18 years of age with RRMM and had received at least two prior lines of therapy including lenalidomide and a PI. Patients demonstrated disease progression during or after completion of their last therapy, had adequate bone marrow function and an Eastern Cooperative Oncology Group (ECOG) performance status (PS) score of ≤2. Prior exposure to pomalidomide and CD38 monoclonal antibody was permitted. Patients were to have measurable disease defined as at least one of the following: serum M protein ≥0.5 g/dL (≥5 g/L), urine M protein ≥200 mg/24 h, or serum free light chain (FLC) assay with involved FLC concentration of ≥10 mg/dL (≥100 mg/L) and an abnormal serum FLC ratio (<0.26 or >1.65).

**Fig. 1:**
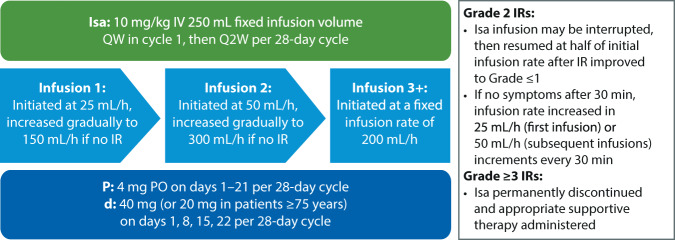
Treatment schedule. *d* dexamethasone, *IR* infusion reaction, *Isa* isatuximab, *IV* intravenous, *P* pomalidomide, *PO* orally, *QW* weekly, *Q2W* every other week.

Patients in Part B were administered isatuximab (10 mg/kg) intravenously (IV) in a fixed-volume infusion of 250 mL, with infusion rate expressed in mL/h. Pd was given at standard doses (pomalidomide 4 mg on days 1–21 and dexamethasone 40 mg [20 mg if ≥75 years] on days 1, 8, 15, and 22 per 28-day cycle). For the first infusion of isatuximab, the initial infusion rate was 25 mL/h. In the absence of IRs after 1 h of infusion, the infusion rate was increased in 25-mL increments every 30 min, to a maximum of 150 mL/h. In the second infusion, the initial infusion rate was 50 mL/h regardless of whether grade ≤2 IRs had occurred during first infusion. In the absence of grade ≥2 IRs after 30 min of infusion, the rate was increased to 100 mL/h for 30 min, then to 200 mL/h for 30 min, and then to 300 mL/h until the total volume was infused. For the third and subsequent infusions, the initial fixed-volume infusion rate was 200 mL/h regardless of whether grade ≤2 IRs had occurred during previous infusions, until the total volume was infused, resulting in a target total infusion time of 75 min (Fig. 1).

In cases of infusion interruption due to grade 2 IRs, isatuximab administration was resumed at one-half of the initial infusion rate after IR improved to grade ≤1. If symptoms did not recur after 30 min, the infusion rate was increased in 25 mL/h (for the first infusion) or 50 mL/h (for subsequent infusions) increments every 30 min, until the total volume was infused. In cases of grade ≥3 IRs, isatuximab administration was permanently discontinued, and appropriate supportive therapy was administered.

All patients received premedication with diphenhydramine 25–50 mg IV (or equivalent), 40 mg dexamethasone IV or orally (or equivalent; 20 mg if ≥75 years), ranitidine 50 mg IV (or equivalent), and acetaminophen 650–1000 mg orally, 15–30 min (but no longer than 60 min) prior to the start of the isatuximab infusion. Dexamethasone was used both as premedication and as part of study treatment. No prophylactic post infusion steroids or bronchodilators were required.

The primary objective was to evaluate the feasibility of isatuximab administered as a fixed-volume infusion in combination with Pd as assessed by the occurrence of grade ≥3 IRs. The primary endpoint was the incidence of grade ≥3 IRs during the first six isatuximab infusions in patients treated for ≥2 cycles. Secondary endpoints were infusion duration, safety profile, immunogenicity, and efficacy. Efficacy endpoints included ORR (complete response [CR] + very good partial response [VGPR] + partial response [PR]), clinical benefit rate ([CBR], CR + VGPR + PR + minimal response [MR]), best overall response, duration of response (DOR), time to first response, PFS, duration of follow-up, and overall survival (OS). Response evaluations were performed by investigators on a monthly basis using the updated International Myeloma Working Group response criteria [30]. Responses (≥PR) and progression were confirmed on two consecutive disease assessments.

The statistical evaluation for all analyses was descriptive and included patients who gave informed consent and received at least one dose (even incomplete) of study treatment. Interphase fluorescent in situ hybridization (FISH) assessment was performed using cytoplasmic immunoglobulin staining followed by FISH (cIg-FISH) from whole bone marrow white blood cells in one central lab. Local FISH results were used when the central lab results were unavailable or inconclusive. An IR adverse event was defined as a treatment-related adverse event occurring within 24 h of each isatuximab administration, however, there was no specific period of time of monitoring after infusions; patients were monitored during isatuximab administration and when clinically indicated. PFS (time from the date of first study treatment administration to the date of first documentation of confirmed progressive disease, symptomatic deterioration, or death), DOR, and OS (time from the date of first study treatment administration to the date of death) were analyzed by the Kaplan–Meier method.

Qualified researchers can request access to patient-level data and related study documents including the clinical study report, study protocol with any amendments, blank case report forms, statistical analysis plan, and dataset specifications. Patient-level data will be anonymized, and study documents will be redacted to protect the privacy of trial participants. Further details on Sanofi’s data-sharing criteria, eligible studies, and process for requesting access are at: https://www.clinicalstudydatarequest.com.

## Results

### Patients

In total, 47 patients were enrolled and treated between March 30, 2018 and December 27, 2018 and all 47 patients received Isa-Pd. The patient baseline characteristics are presented in Table 1. All patients had previously received lenalidomide and 48.9% had received prior pomalidomide treatment. All 23 patients who received prior pomalidomide were refractory to it. Prior daratumumab exposure was reported for 14.9% of patients; prior elotuzumab exposure was reported for 19.1% of patients. All patients with prior daratumumab exposure were refractory to daratumumab; none of the patients received daratumumab as last regimen, six out of seven also received prior pomalidomide. At the time of data cut-off (November 18, 2019), 22 patients (46.8%) remained on treatment and 25 patients (53.2%) had discontinued treatment. Of the 47 patients, 15 (31.9%) discontinued because of disease progression, five (10.6%) because of adverse events, and five (10.6%) because of other reasons. One patient (2.1%) prematurely discontinued pomalidomide treatment due to an adverse event, and no patient prematurely discontinued dexamethasone treatment.

**Table 1:** Patient demographics and disease characteristics.

Characteristic	All patients (*N* = 47)
Median age, years (range)	65 (45–85)
Median time since initial diagnosis, years (range)	6.2 (1.1–22.7)
Gender, male, *n* (%)	27 (57.4)
Race, *n* (%)	
White	42 (89.4)
Black or African American	3 (6.4)
Asian	1 (2.1)
Other	1 (2.1)
ISS stage at study entry, *n* (%)	
I	23 (48.9)
II	12 (25.5)
III	7 (14.9)
Unknown	5 (10.6)
ECOG performance status, *n* (%)	
0	5 (31.9)
1	30 (63.8)
2	2 (4.3)
Respiratory disorders at baseline, *n* (%)	
Asthma	8 (17.0)
Bronchial hyperreactivity	1 (2.1)
COPD	2 (4.3)
At least 1 transplant, *n* (%)	32 (68.1)
Autologous stem cell transplant	31 (66.0)
Allogenic stem cell transplant	2 (4.3)
Number of prior lines^a^, median (range)	3 (1–8)
Prior treatments	
Lenalidomide	47 (100)
Pomalidomide	23 (48.9)
Bortezomib	46 (97.9)
Carfilzomib	11 (23.4)
Daratumumab	7 (14.9)
Elotuzumab	9 (19.1)
Refractory status, *n* (%)	
Last regimen	41 (87.2)
Lenalidomide	41 (87.2)
Pomalidomide	23 (48.9)
Bortezomib	26 (55.3)
Carfilzomib	7 (14.9)
Daratumumab	7 (14.9)
Elotuzumab	9 (19.1)
Immunomodulatory drug, proteasome inhibitor, and daratumumab	7 (14.9)
Lenalidomide, pomalidomide, bortezomib, carfilzomib, and daratumumab	2 (4.3)
High-risk cytogenetics^b^, *n* (%)	10 (21.3)
Median number of cycles received^c^ (range)	9 (1–19)
Overall median duration of exposure, weeks (range)	36.9 (1–77)

*COPD* chronic obstructive pulmonary disease, *ECOG* Eastern Cooperative Oncology Group, *ISS* International Staging System

^a^1 patient (2.1%) received 1 prior line of treatment and 17 patients (36.2%) received 2 prior lines of treatment.

^b^Either del(17p), or t(4;14), or t (14;16).

^c^31 patients (66.0%) started at least 6 cycles and 18 patients (38.3%) started at least 12 cycles.

The median age was 65 years. All patients had an ECOG PS of 0 or 1, except two patients (4.3%) who had an ECOG PS of 2. The median number of prior treatment lines was three (range, 1–8) with one patient (2.1%) having received one prior line of treatment and 17 patients (36.2%) having received two prior lines of treatment. All patients had received an IMiD, a PI, and corticosteroid in prior lines of treatment. There were 10 patients (21.3%) with high-risk cytogenetic characteristics, including del(17p) in seven patients (14.9%), t(4;14) in three patients (6.4%), and t(14;16) in one patient (2.1%). Most patients (33 patients, 70.2%) had bone lesions at baseline, and 12 (25.5%) patients had soft-tissue plasmacytoma present at baseline. The median number of cycles of study treatment received was nine (range, 1–19), with 31 patients (66.0%) having started at least 6 cycles and 18 patients (38.3%) having started at least 12 cycles. The overall median duration of exposure was 36.9 weeks (range, 1–77).

### Safety

**Fig. 2:**
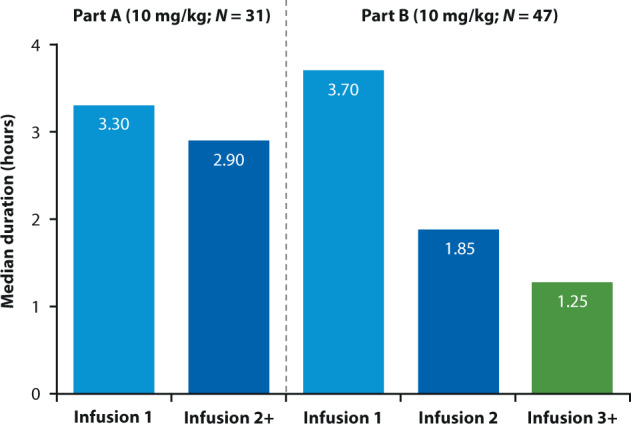
Fixed-volume infusion of isatuximab. Duration of isatuximab infusion, by infusion number for Part A (weight-based infusion volume; mg/h) and Part B (fixed-volume infusion; mL/h).

The primary endpoint was the incidence of grade ≥3 IRs during the first six isatuximab infusions in patients treated for ≥2 cycles. Overall, IRs of any grade were reported in 19 patients (40.4%), and in 20 episodes of 871 infusions (2.3%). There were no grade ≥3 IRs. IRs were all grade 2 in severity, occurred only during the first isatuximab infusion, and recovered on the day of onset. Of note, in Part A, 48.3% of patients who received isatuximab 10 mg/kg experienced an IR, including one (3.2%) with grade 3 IRs [29]. IRs were managed with dose interruption in 18 patients (38.3%), while the dose was not interrupted in one patient (2.1%). Additional medications were administered to 17/19 patients (89.5%) experiencing an IR, and consisted of one or more of the following: H1/H2 blockers, acetaminophen, montelukast, steroids, and bronchodilators. Of the seven patients with prior exposure to daratumumab, three experienced IRs. Across all treated patients, the median duration of the first infusion was 3.7 h (range, 1.0–6.1 [222 min; range, 60–366]), 1.85 h (range, 1.5–3.9 [111 min; range, 90–234]) for the second infusion, and 1.25 h (range, 0.8–3.4 [75 min; range, 48–204]) for 3+ infusions (Fig. 2). A post-hoc analysis comparing the duration of infusion for weight-based volume (Part A) to that of fixed-volume isatuximab infusion (Part B) demonstrated that the duration of infusion for fixed volume is significantly longer at first infusion, but significantly shorter from second infusion onwards (Table 2).

**Table 2:** Duration of infusion in the all treated population: weight-based volume infusion (Part A) versus fixed-volume infusion (Part B).

Median duration of infusion, h^a^	Isa-Pd (10 mg/kg)		
	Part A [29] *N* = 31	Part B *N* = 47	Two-sided *p*-value^b^
1st Infusion	3.32	3.70	0.0007
2nd Infusion	2.88	1.85	<0.0001
3rd Infusion	2.89	1.25	<0.0001
4th Infusion	2.71	1.25	<0.0001
5th Infusion	2.67	1.25	<0.0001
6th Infusion	2.81	1.25	<0.0001
≥2nd Infusion	2.87	1.25	<0.0001
≥3rd Infusion	2.87	1.25	<0.0001

*d* dexamethasone, *h* hours, *Isa* isatuximab, *P* pomalidomide

^a^Duration of infusion is defined from the start time of infusion to the end time of infusion including interruption time (if any).

^b^A Mann–Whitney *U* test has been performed.

All patients had at least one treatment-emergent adverse event (TEAE; any grade) and 35 patients (74.5%) had grade ≥3 TEAEs, regardless of relationship to study treatment (Table 3). Five patients (10.6%) discontinued treatment because of TEAEs; one patient (2.1%) experienced TEAEs leading to premature discontinuation of pomalidomide. The most common non-hematologic TEAEs of any grade were fatigue (63.8%), IRs, cough, and upper respiratory tract infection (40.4% each). In addition, 70.2% of patients had grade 3 or 4 laboratory neutropenia (34.0% and 36.2%, respectively). Febrile neutropenia was reported in nine patients (19.1%), with 4.3% being grade ≥3. The most frequently reported grade ≥3 non-hematologic TEAE was pneumonia (five patients [10.6%]). Treatment-related TEAEs were experienced by 45 patients (95.7%), with 28 (59.6%) experiencing treatment-related TEAEs of grade ≥3. Serious TEAEs were observed in 27 patients (57.4%), which were treatment-related in 14 patients (29.8%). Six patients (12.8%) died within 30 days from their last study treatment administration, three because of adverse events not related to study treatment (acute myocardial infarction, sepsis, and rectal hemorrhage and sepsis), two because of disease progression, and one because of other reasons (sudden death, adjudicated as not related to study drug). Five patients (10.6%) discontinued study treatment due to TEAEs. In addition to the three patients with fatal events described above, there was one patient with serious grade 3 spinal cord compression, which was considered not related to study treatment. One patient selectively discontinued pomalidomide, while continuing treatment with isatuximab and dexamethasone, because of non-serious grade 1 events of tremor, gait disturbance, and flushing.

**Table 3:** Most common TEAEs occurring in ≥20% of patients, all grades.

TEAE	Isa-Pd *N* = 47	
	All grades *n* (%)	Grade ≥ 3 *n* (%)
Any	47 (100)	35 (74.5)
Fatigue	30 (63.8)	2 (4.3)
Infusion reactions	19 (40.4)	0
Cough	19 (40.4)	0
Upper respiratory tract infection	19 (40.4)	3 (6.4)
Neutropenia	18 (38.3)	18 (38.3)
Diarrhea	16 (34.0)	2 (4.3)
Dyspnea	16 (34.0)	2 (4.3)
Nausea	16 (34.0)	0
Insomnia	15 (34.0)	1 (2.1)
Back pain	14 (29.8)	2 (4.3)
Constipation	14 (29.8)	1 (2.1)
Arthralgia	13 (27.7)	3 (6.4)
Peripheral sensory neuropathy	10 (21.3)	3 (6.4)
Pneumonia	10 (21.3)	5 (10.6)

*d* dexamethasone, *Isa* isatuximab, *P* pomalidomide, *TEAE* treatment-emergent adverse event

Hematologic laboratory abnormalities of anemia and decreased lymphocyte and white blood cell counts (all grades) were reported in all 46 (100.0%) patients. Grade ≥3 hematologic laboratory abnormalities were common (Table 4). Grade 3 and grade 4 neutropenia was reported in 34.8% and 37.0% of patients, respectively. Grade 3 anemia was reported in 21.7% of the patients, and no patient reported grade 4 anemia.

**Table 4:** Grade 3–4 hematologic laboratory abnormalities.

Hematologic laboratory abnormality^a^, *n* (%)	Isa-Pd *N* = 46^b^	
	Grade 3	Grade 4
Lymphocyte count decreased	25 (54.3)	5 (10.9)
White blood cell decreased	24 (52.2)	7 (15.2)
Neutrophil count decreased	16 (34.8)	17 (37.0)
Anemia	10 (21.7)	0
Platelet count decreased	9 (19.6)	4 (8.7)

*d* dexamethasone, *Isa* isatuximab, *P* pomalidomide

^a^Hematological laboratory abnormalities were assessed during the study (Table 2) and were recorded as TEAEs only if they were serious or led to study treatment modification or discontinuation.

^b^The number of patients who had that parameter assessed post-baseline (not missing) during the TEAE period.

### Efficacy

At a median follow-up duration of 9.9 months (range, 0–17.3), the ORR was 53.2% (95% CI, 38.1–67.9), including 12 PRs, 11 VGPRs, and 2 CRs. The CBR was 72.3%. The ORR was 60.0% for patients without prior daratumumab exposure and 14.3% (one patient) in seven patients who had previous exposure to daratumumab. In six patients evaluable for response who had prior daratumumab exposure, one had PR, two had MR, and three had stable disease. The ORR in patients with prior pomalidomide treatment and those without prior pomalidomide or daratumumab was 52.2% (12 of 23 patients) and 56.5% (13 of 23 patients), respectively. Of the 13 patients who had received >3 prior lines of therapy, two had VGPR and four had PR. In the 10 patients with high-risk cytogenetics, the ORR was 40.0% (95% CI, 12.2–73.8), including one PR, two VGPRs, and one CR.

The median time to first response was 0.95 months (range, 0.9–3.4) and the median time to best response was 1.3 months (range, 1.0–8.3). In 25 patients, DOR was assessed and 21 responding patients who had an ongoing response were censored. Median DOR has not been reached. At the time of analysis, 20 patients (42.6%) were reported to have had a PFS event, and 27 patients (57.4%) were censored. The median PFS has not been reached; the 6-month probability of PFS was 65.0% (95% CI, 49.3–76.9) and the 12-month probability was 55.7% (95% CI, 40.1–68.8). Likewise, the median OS has not been reached; the 6-month probability of survival was 84.5% (95% CI, 70.1–92.3) and the 12-month probability of survival was 70.6% (95% CI, 53.7–82.3).

## Discussion

This was a phase 1b study comprising two parts, of which the objective of Part B was to evaluate the feasibility, safety, and efficacy of isatuximab 10 mg/kg administered with a 250 mL fixed-volume infusion (rate in mL/h) in combination with Pd in RRMM, with the aim of reducing the infusion time starting with the second infusion. Overall, this combination was well tolerated, with no grade ≥3 IRs observed. All IRs were grade 2, occurred during the first infusion, and resolved on the same day. Because of the risk of IRs inherent with monoclonal antibody infusion, premedications were administered to minimize this risk, and the infusion rate was increased during the first infusions. No prophylactic post infusion steroids or bronchodilators were required. The median infusion time for isatuximab decreased from 3.7 h during the first infusion to 1.85 h during the second infusion, and to 1.25 h (75 min) for 3+ infusions. This is considerably shorter than the infusion time from Part A of this study when isatuximab was administered as mg/hour, with the first and subsequent infusions lasting 3.3 and 2.9 h, respectively [29]. The safety and efficacy data with fixed-volume infusion are consistent with those observed with the infusion schedule used in Part A of the study. Both reported 19 patients with IRs (Part B: 40.4% of patients; Part A: 42.2% of patients). The ORR in all subjects who received any study treatment on Part B was 53.2% and 62.2% in Part A, with similar VGPR and CR rates (23.4% and 4.3% in Part B versus 22.2% and 2.2% in Part A, respectively). Responses were durable, with the median DOR not reached in Part B compared with 18.7 months in Part A.

The safety profile of Pd in combination with isatuximab administered as fixed-volume infusion on this study appears to be consistent with the safety profile of the pivotal ICARIA-MM study (NCT02990338) [6]. The median number of cycles patients received in this trial was 9, compared with 10 cycles in the ICARIA-MM study. IRs were similar at 40.4%, compared with 38.2% in the ICARIA-MM study. Grade ≥3 TEAEs and serious AEs (74.5% and 57.4%, respectively) were slightly lower in this trial compared with the ICARIA-MM study (86.8% and 61.8%, respectively). Similarly, TEAEs leading to treatment discontinuation or death were consistent in this study (10.6% and 12.8%, respectively), compared with the ICARIA-MM study (7.2% and 7.9%, respectively). Median DOR, PFS, and OS data appear very promising, however, these data are still maturing, with a median duration of follow-up of 9.9 months and 46.8% of patients still on treatment. Nonetheless, these preliminary efficacy data so far resemble those observed in the ICARIA-MM study. The ORR in patients not exposed to daratumumab was 60.0% compared with 60.4% in ICARIA-MM. The 1-year probability of PFS and OS were 55.7% and 70.6%, respectively, compared with 47.6% and 72.0% in ICARIA-MM.

Patients in the current study had been heavily pretreated, with a median of 3 prior lines of therapy, with some patients receiving up to 8 prior lines of therapy, and all patients were previously treated with a PI and an IMiD. In addition, most were refractory to the current standard of care therapies (lenalidomide: 87.2%; PIs: 74.5%; and both IMiDs and PIs: 74.5%). Likewise, 21.3% of the patient population had high-risk cytogenetic characteristics. The presence of high-risk cytogenetic abnormalities is associated with reduced survival of patients with CD38 antibody-refractory MM [31]. Although the patient numbers are small in this study, the robust response rate (40.0%) in this group is promising, and especially given the relapsed and refractory setting in which patients were multi-agent resistant [32]. In the ICARIA-MM trial, the observed PFS benefit in patients treated with Isa-Pd was maintained across patients with high-risk cytogenetics and was similar to patients with standard risk cytogenetics (hazard ratio [HR], 0.66 [95% CI, 0.33–1.28] and HR 0.62 [95% CI, 0.42–0.93], respectively) [6]. Also, of note, the ORR was 14.3% and CBR was 42.9% in the seven patients who had previous exposure to daratumumab, compared with 60.0% and 77.5% for patients without prior daratumumab. Despite the small sample, this suggests that the Isa-Pd combination is less active in patients with prior daratumumab exposure. Daratumumab treatment has been linked to a reduction in CD38 expression levels on MM cells within hours after treatment start [33], but further mechanistic investigations are required to dissect this highly clinically relevant issue.

The results from Part B of this two-part, phase 1b study confirm the safety, efficacy, and feasibility of isatuximab administered by a fixed-volume infusion method. Efficacy and safety were consistent with Part A of this study and the pivotal ICARIA-MM study. The fixed-volume infusion administration of isatuximab reduced time of infusion by >60 min for the second infusion and >90 min for subsequent infusions, compared with the weight-based infusion of isatuximab in Part A. The results of this study led to the approval of Isa-Pd accelerated infusion and serves as the basis for how standard of care isatuximab is dosed, as described in the United States Prescribing Information and the European Union Summary of Product Characteristics. These reduced infusion times for isatuximab are the shortest IV infusion times of any approved anti-CD38 monoclonal antibody [26, 27, 34, 35], thus improving patient convenience while maintaining safety and simplifying infusion schedules to reduce administration errors, thus facilitating real-world practice [36]. The combination of daratumumab IV with Pd has twice the infusion time compared with Isa-Pd and is currently approved in the United States but not Europe. A subcutaneous (SC) formulation of daratumumab (DARZALEX FASPRO™, daratumumab co-formulated with hyaluronidase-fihj) was recently approved in the United States and in Europe; however, not in combination with Pd. A SC formulation of isatuximab is being developed and tested in combination with Pd in a phase 1b study (ClinicalTrials.gov, NCT04045795). These data support the use of isatuximab 10 mg/kg administered with a 250 mL fixed-volume infusion, with a target total infusion time of 75 min in combination with Pd in heavily pretreated RRMM patients.
